# Platelet Metabolism and Other Targeted Drugs; Potential Impact on Immunotherapy

**DOI:** 10.3389/fonc.2018.00107

**Published:** 2018-04-20

**Authors:** Preeti Kanikarla-Marie, Michael Lam, Alexey V. Sorokin, Michael J. Overman, Scott Kopetz, David G. Menter

**Affiliations:** Department of GI Medical Oncology, The University of Texas MD Anderson Cancer Center, Houston, TX, United States

**Keywords:** platelets, cyclooxygenase, platelet inhibitors, non-steroidal anti-inflammatory drugs, aspirin, immunotherapy

## Abstract

The role of platelets in cancer progression has been well recognized in the field of cancer biology. Emerging studies are elaborating further the additional roles and added extent that platelets play in promoting tumorigenesis. Platelets release factors that support tumor growth and also form heterotypic aggregates with tumor cells, which can provide an immune-evasive advantage. Their most critical role may be the inhibition of immune cell function that can negatively impact the body’s ability in preventing tumor establishment and growth. This review summarizes the importance of platelets in tumor progression, therapeutic response, survival, and finally the notion of immunotherapy modulation being likely to benefit from the inclusion of platelet inhibitors.

## Introduction

Platelets arise from immune stem cell lineages in the bone marrow that are critically important in blood clotting and wound healing (1, 2). They are generated as cell fragments formed by membrane bound blebs at the surface of megakaryocytes. Platelets actively participate in the hemostasis, wounding, and immune processes required for normal functioning of the body (1, 2). In instances where the homeostasis is deregulated or dysfunctional, platelet functions can become aberrant. At times, even the thrombi formed for the resolution of the wounds may cause thromboembolic complications (3). Cancer is one such disease that can disrupt the normal functioning and production of platelets which in part is contributed by the pro-carcinogenic inflammatory milieu. There has been a long-standing link between cancers, thromboembolism, and thrombocytosis (4–6). Abnormalities in the number of platelets are most commonly observed in several cancers: colorectal, ovarian, and breast being some of them. Colorectal cancer (CRC) is one such type that is frequently associated with thrombocytosis with significant correlations for worse overall survival and recurrence rates (5, 7, 8). High platelet counts in these patients also correlated with tumor invasiveness, metastasis, and worse survival outcomes (9–13).

It appears that platelets not only increase in numbers and get activated with cancer progression but also return to their normal levels and functional state following cancer therapy. These features of platelets can be tested in a relatively inexpensive and non-invasive manner using routine blood profiles and may serve as prognostic and diagnostic biomarkers of cancer. As a patient’s disease progresses through the advanced stages of CRC, they can exhibit an increase in platelet activation with increased platelet factors and surface markers of activation (14–18). There are also reports showing that anticancer therapy could induce changes in the activation of platelets, their function, as well as their morphology (19). In the case of ovarian cancer, platelets were shown to increase the growth of cancer cells *in vitro* and *in vivo* animal models of cancer. Platelet alterations and thrombogenesis were seen in ovarian cancer patients (20–25). Mechanistically, IL6 production by an ovarian tumor can stimulate thrombopoietin production in the liver that elevates platelet production in the bone marrow (24). Platelets from ovarian cancer patients may also carry pro-coagulatory signatures based on their lipid profiles (26). The role of platelets in reducing cell death and enabling metastasis was also shown by activating YAP1 signaling in ovarian cancer (27). Similarly, many other reports show an active involvement of platelets in tumorigenesis and metastasis (28–32).

## Platelet Activation and Platelet Counts in Cancer

Reactive platelets can recruit more platelets to form platelet aggregates and can also engage in heterotypic aggregates with leukocytes (33). Platelets upon activation release granules and extracellular vesicles that are rich in proteins, mRNA, miRNA, and lipids. These loaded particles can be involved in the transfer of receptors to the surface of other cells, including but not limited to lymphocytes, macrophages, and tumor cells by membrane fusions, and can also induce gene expression changes in the target cells by activating transcription factors (34–37). The transfer of cargo is not always unidirectional, as platelets that are in the vicinity of the tumor site can also take up RNA and other molecules from the tumor. The platelets that have undergone modifications after interacting with the tumor are termed as tumor-educated platelets (TEP). Recently, these TEPs have been explored for their significance and in extracting tumor-specific information (38). The reliability of using platelets to detect normal vs. tumor-educated platelet, along with the possible prediction of primary tumor location, all based on the platelet gene expression profiles has been successfully shown (39–42). The apparent benefits of such analyses involving platelet isolation could be diverse. The key feature of such tests is the ease of platelet isolation. Non-invasive blood-based liquid biopsies could be advantageous in early detection and screening of cancer. The importance of increased mean platelet volume, platelet counts, size, and platelet to lymphocyte ratio indexes in individuals has already been well recognized in predicting poor outcomes as well as in predicting association in diabetes, cerebral, and cardiovascular events (43–48). The same indices also may be predictive regarding cancer prognosis, treatment response outcomes, and overall survival analysis (49–55). An elevated platelet distribution width-to-platelet count ratio was shown to significantly reduce disease free survival in patients with breast carcinoma (52). As blood draw procedures are already in place and routinely used in cancer studies in a prospective or a retrospective fashion, the added benefit of achieving platelet-related tumor-specific signature as described by Best et al., and the treatment outcomes could become a standard for cancer screening and diagnosis (38–40, 56).

## Platelets, Serum Growth Factors, and Platelet-Rich Plasma Therapy

Normal platelets in circulation range in number between 150,000 and 400,000/μl. Based on sheer numbers and biologic properties there are many opportunities to engage in multiple aspects of tumor formation. In particular, it has long been known that platelets provide the bulk of the serum factors that promote cell growth, which is routinely used in culturing cells (1, 57–59). Platelet release reactions factors not only provide growth factors but also metalloproteinases involved in tissue reorganization (60–62) and have found clinical use in platelet-rich plasma therapy (60, 63–66).

## Platelets and Circulating Tumor Cells (CTCs)

Once within the blood stream, tumor cells that enter the circulation are known as CTCs. These cells can become clinically evident when an established tumor starts to shed off cells from its bulk, or by other means such as sloughing, or even by active entry (intravasation) of cancer cells into abnormal tumor blood vessels (67–71). These are immediately sensed by the large number of platelets in the circulation and perivascular microenvironment (72–76). The cross talk between platelets and tumor cells leads to the rewiring of platelets as they become tumor-educated platelets. This interaction can result in the formation of heterotypic aggregates along with the release of growth promoting factors and the entrapment of the tumor cells with platelet aggregates in the microvasculature (1, 2). Platelets also have an active cytoskeleton enabling their unrestricted movements (77–79). In addition, properties such as the lack of a nucleus, discoid shape, and small size are ideal for platelets to migrate into extravascular tissues easily supporting the invasion of CTCs and their metastasis (1, 24, 80–84). There is also evidence of platelets shielding the CTCs and protecting them from immune surveillance and elimination (85). The selective survival advantages that tumor cells gain from interacting with platelets enables them to withstand or evade immune attacks, take advantage of enhanced access to platelet released-growth factors. These interactions also benefit from relatively easy passage of platelets that invade unencumbered by a cell nucleus as they migrate into surrounding tissues due to their small size and active cytoskeleton. Then once in the perivascular spaces, factors released by platelets stimulates tumor cells to home, extravasate, and metastasize. Accordingly, a high probability exists that targeting or suppressing these tumor cell and platelet interactions would yield beneficial results. As one might expect, inhibiting this interaction of tumor cells with platelets was shown to hinder tumor cell survival, growth, and metastasis in experimental cancer models and had significance in clinical trials (86–94).

## Tyrosine Kinase Inhibitors and the Platelet Connection

The release of alpha granules leads to local increases in multiple growth factors and tyrosine kinase receptor activating molecules. Among the growth stimulating and vascular permeability regulating molecules like platelet-derived growth factor (PDGF) that are inhibited by a number of tyrosine kinase receptor inhibitors and may be influenced by targeted therapy [(95, 96); Table 1]. Likewise, vascular endothelial growth factor (VEGF) receptors and their inhibitors can be influenced by inhibitors that involve platelet alpha granule releasates (97–99). These factors affect endothelial cells, pericytes, lymphocytes, and tumor cells. They influence vascular permeability, angiogenesis, inflammation, and immune responsiveness. In addition to use in chemotherapy (95–99), there is renewed interest in these PDGFR and VEGFR targeted compounds in combination with checkpoint blockade inhibitors (100). Similarly, other immunoreceptor-based therapies can have a direct effect on FcgammaRIIa tyrosine kinase receptors in platelets. For example, BCR-ABL inhibitor ponatinib inhibits platelet immunoreceptor tyrosine-based activation motif (ITAM) signaling under shear which includes platelet activation and aggregate formation (101, 102). Overall, TKIs could potentially be reconsidered in the context of platelet function during immunotherapy.

**Table 1:** Platelet targeted therapeutics.

Panel	Inhibitor type	Inhibitor	Target pathway
A	vonWillebrand GPIa/IIa	Hookworm secretion	Surface receptor
	Collagen GPVI	Revacept	Surface receptor
	Focal adhesion kinase	GSK2256098, PF-562271, Defactinib, PF-573228, Y15 and Y11, CEP37440	Kinase pathway
B	GPIIb/IIIa	Abciximab, eptifibatide, tirofiban, XV454, heparins	Surface receptor
	Vitronectin αv/β3	SB-273005, SC-68448, vitaxin	Surface receptor
C	P-Selectin	Rivipansel, crizanlizumab, heparins	Surface receptor
	CLEC-2	2CP, Mabs	Surface receptor
	ROCK	Y27632	Signal transduction
	Myosin II	Blebbistatin	Signal transduction
	PAR 1 and PAR 4	Voraxopar, SCH 79797, RWJ56110	G_q_-protein-coupled receptor
D	PDGFR	Imatinib, sorafenib, sunitinib, nilotinib, dasatinib, axitinib, cediranib, regorafinib, pazopanib, midosotaurin	Tyrosine kinase receptor pathway
	VEGFR	Bevecizimab, aflibercept, ramucirumab, sunitinib, lenvatinib, vandetanib, sorafinib, pazopanib HCL, axaitinib, regroafinib, cabozantinib	Tyrosine kinase receptor pathway
	FcgammaRIIa	Ranibizumab or bevacizuma	Tyrosine kinase receptor pathway
E	ADP receptor, P2Y_1_	A2P5P, MRS2179, A3P5P, MRS2500	G_αq_-protein-coupled receptor
	ADP receptor, P2Y_12_	Clopidogrel, ticlopidine, prasugrel, ticagrelor, cangrelor, elinogrel	G_i_-protein-coupled Receptor
F	Serotonin receptor 5HT_2A_R	ADP-791, naftidrofuryl, sarpogrelate, AT-1015	G_αq_-protein-coupled receptor
G	Thromboxane A_2_ (TxA_2_) receptor α or β	Terutroban, daltroban, picotamide, sulotroban, CAY10535, ifetroban, SQ 29,548, BM 567, pinane TxA_2_	G_12/13_-protein-coupled receptor
	Thromboxane A_2_ Synthase	OKY-046, ridogrel	TxA_2_ synthesis enzyme
	Cyclooxygenase 1 (COX-1)	Aspirin, ASP6537, SC560, FR122047, mofezolac, fluorofuranones	Prostaglandin H_2_ synthesis enzyme (substrate for all PG synthesis)
H	Prostaglandin E_2_ (PGE_2_) receptor EP3	DG-041	G_αq_-protein-coupled receptor
	12-Hydroxyeicosa tetraenoic acid (12-HETE) Receptor, GPR31	ML355, NCTT-956	G-protein-coupled receptor
I	Prostacyclin (PGI_2_) receptor	PGI_2_ agonist endogenous platelet inhibitor, iloprost, treprostinil, selexipag	G_αs_-protein-coupled receptor
	Phosphodiesterase (PDE) PDE3 and PDE5	Cilostazol, dipyridamole, sildenafil	Phosphodiesterase enzyme inhibitor

**Figure 1:**
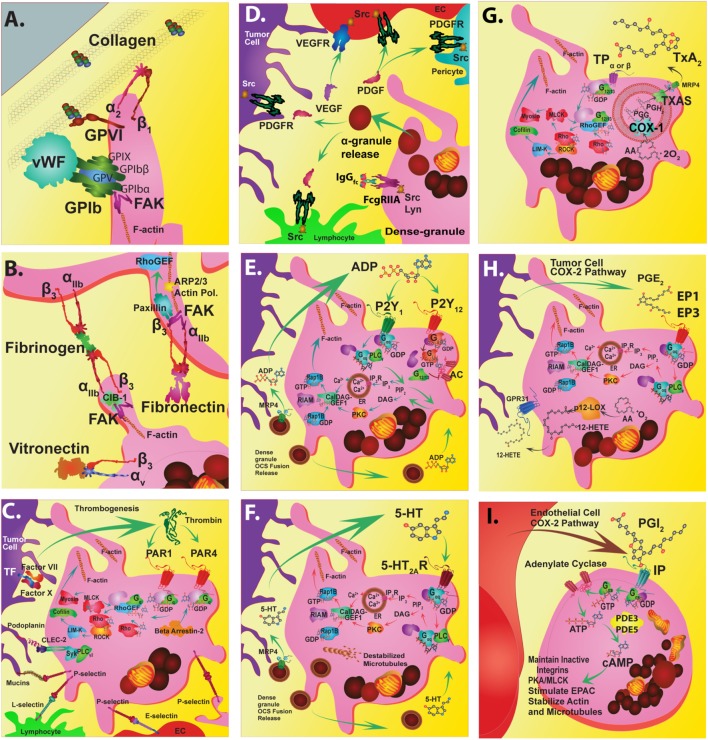
Platelet targeted therapeutics. The platelet plasma membrane displays multiple receptors that can interact with agonists, antagonists, matrix proteins, collagen, other platelets, endothelial cells, immune cells, and tumor cells. **(A)** Platelets adhere to the damaged vascular endothelium via GPIb-IX-V complex to von Willebrand factor (vWF) and *via* GPVI and GPIa/IIa (α_2β1_) to collagen. **(B)** Focal adhesion kinase (FAK) helps mediate GPVI binding to collagen among other integrin-mediated interactions. FAK inhibitors could also potentially inhibit GPIIb/IIIa (α_IIbβ3_) interactions that stimulate calcium and integrin-binding protein 1 (CIB-1) or paxillin that is linked to Rho guanine nucleotide exchange factor (Rho-GEF) signaling. Alternatively, FAK inhibition may also alter actin-related protein 2/3 complex interactions during actin polymerization and shape change. Direct inhibition of GPIIb/IIIa interactions with fibrinogen or fibronectin during platelet aggregation can be disrupted by receptor antagonists. Similarly, direct inhibition of a_v_b_3_ interactions with vitronectin can also be inhibited. **(C)** Thrombin G_q_-protein-coupled receptors involved in platelet activation are protease-activated receptors (PAR) 1 and 4. PARs are stimulated by tumor cell tissue factor-factor VII-factor X complex. Thrombin stimulation of PAR 1 acts through the Rho-GEF pathway while PAR4 G_q_ activation occurs through beta arrestin-2. Signal transduction targets include Rho-associated kinase (ROCK) or the cytoskeletal protein myosin II. Tumor cell podoplanin interacts with platelet C-type lectin domain family 2 (CLEC-2) that transduce signals through spleen tyrosine kinase (Syk) and phospholipase C gamma2 (PLCγ_2_). Tumor cell mucins and other carbohydrate moieties interact with P-selectin are also targets. These P-selectin targets include interactions with lymphocyte L-selectins or endothelial cell E-selectins. **(D)** The release of alpha granules leads to localized increases in growth factors such as platelet-derived growth factors (PDGFs) and vascular endothelial cell growth factor (VEGF) and tyrosine kinase receptor stimulating molecules such as fc receptor stimulating molecules FcgammaRIIa. **(E)** The activation of platelets by ADP (adenosine diphosphate) released from dense granules mainly involves P2Y_1_ or P2Y_12_ receptors. P2Y_1_ signals through G_ag_-protein-coupled receptors that stimulate PLCγ followed by phosphatidylinositol 4,5-bisphosphate (PIP_2_) and inositol trisphosphate 3 (IP_3_) that stimulates its receptor embedded in the endoplasmic reticulum (ER), which causes calcium ion release (Ca^2+^). Alternately, diacylglycerol (DAG) interacts with protein kinase C (PKC). These interactions impinge upon DAG-regulated guanine nucleotide exchange factor I (CalDAG-GEFI)-Ras-related protein 1 (Rap1) releasing Rap1-GTP-interacting adaptor molecule (RIAM) leading to actin changes. **(F)** Serotonin (5-hydroxytryptamine) is also released from dense granules that act through 5-hydroxytryptamine receptors (5HT_2A_R) that activate the G_aq_ pathway and Ca^2+^ release. **(G)** An important antiplatelet agent is aspirin that is well known to prevent cancer progression. Aspirin irreversibly acetylates cyclooxygenase 1 (COX-1) eliminating all prostaglandin (PG) synthesis. COX-1 enzymatically adds two oxygens to arachidonic acid to produce PGG_2_ and then PGH_2_, which is converted to various PGs by synthase enzymes. Key platelet PGs are the potent pro-aggregatory agent thromboxane (TX)A_2_ synthesized by TXA_2_ synthase. **(H)** Also, PGE_2_ synthesized by PGE_2_ synthase. TXA_2_ and PGE_2_ cause different platelet responses by stimulating various isoforms of G-protein-coupled TP or EP receptors. TP signals through G_12/13_ and Rho-GEF followed by Rho-associated kinase (ROCK), LIM domain kinase (LIMK), and cofilin and subsequent interactions with actin. Additional interactions include those with myosin light chain kinase followed by myosin. Similarly, EP_3_ receptors stimulate the same signal transduction pathways as G_aq_-calcium release linked receptors. **(I)** An important G_as_-protein-coupled receptor is the IP for prostacyclin (PGI_2_) that prevents aggregation by stimulating cyclic adenosine monophosphate (cAMP) production by adenylate cyclase (AC) and is influenced by phosphodiesterase 3 or 5 activity. Another abundant eicosanoid produced from arachidonic acid by platelets is 12(S)-HETE [12(S)-hydroxyeicosatetraenoic acid] *via* the activity of the platelet-type lipoxygenase (p12-LOX). Recently, 12-(S)HETE is proposed to activate orphan receptor GPR31.

*This table summarizes the platelet-related targets, inhibitors, and pathway targets dipicted in Figure 1*.

## Platelet Metabolism Targeted Non-Steroidal Anti-Inflammatory Drug (NSAID) Use in Cancer

Given the importance of platelets in almost all aspects and stages of cancer progression, Table 1 highlights platelet inhibition pathways. The inhibition of platelets is receiving renewed attention as a target. Interest in inhibiting platelets heightens in the case of cancers where the disease risk increases with chronic inflammation. There have been a number of clinical trials addressing the role of platelets in cancer patients. The most commonly used drugs to target platelets are the NSAIDs. NSAIDs such as aspirin, rofecoxib, sulindac, and celecoxib are effective in reducing cancer but have potential cardiovascular risks (103–106). One of the most heavily used drug on the market is aspirin, it irreversibly acetylates platelet cyclooxygenase 1 (COX-1), which is the rate limiting NSAID eicosanoid metabolic target found in platelets (Figure 1). By contrast, aspirin less selectively inhibits cyclooxygenase 2 (COX-2), expression, which is commonly upregulated in cancer (107). Despite gastrointestinal side effects that may be heightened in patients with preexisting gastric lesions, aspirin has been used in prevention studies as well as in experimental models of cancer to inhibit cancer cell growth, platelet-tumor cell interactions, heterotypic aggregate formation, platelet facilitated tumor cell invasion, and metastasis (108–113). In general, the risk/benefit of using aspirin is favorable over a given lifetime (114). Moreover, aspirin has also been used in combination therapies. In one study, breast cancer patients were given aspirin along with tamoxifen therapy, while in another study they were given aspirin along with clopidogrel in an effort to reduce the release of platelet proteins and the number of CTCs, respectively. Though the impact of platelet inhibition on CTC did not correlate with the platelet inhibition, aspirin therapy was favorable in reducing the release of platelet-angiogenic proteins (94, 115). Another phase II trial with metastatic cancer patients reported that aspirin treatment significantly reduced CTC numbers in metastatic CRC but did not have significance in metastatic breast cancer (116).

Epidemiological evidence also suggests that aspirin (and other NSAIDs) alone or in combination is beneficial in chemo prevention. In one such study, molecular pathological analyses conducted on 964 CRC patients suggested that the PIK3CA mutation in CRC may be used as a biomarker in identifying patients that could potentially receive aspirin as adjuvant therapy (117). The study provided association between mutated PIK3CA and longer survival in patients who received aspirin after cancer diagnosis. This association was lost in patients with wild-type protein (117). In other secondary epidemiological analyses, evidence from cardiovascular disease prevention studies suggests that aspirin therapy reduces CRC incidence and even mortality after 10 years of use (118–121). Several reports from literature also support that aspirin (and other NSAIDs) has proven to be effective in experimental models of cancer as well as in patient studies related to colorectal, breast, and ovarian cancers (94, 110–112, 115, 121–129). However, aspirin gastrointestinal toxicity potentially limits its use as a chemopreventive. To balance the risk versus the benefit of administering chemopreventive interventions such as aspirin, the US Preventive Services Task Force has made recommendations based on clinical evidence (130). The recommendations are directed toward primary prevention of cardiovascular disease and CRC in adults aged 50–59 years of age, particularly those at high risk for disease with the exception of individuals who are at increased risk of bleeding (130, 131). The benefits of cancer prevention with NSAID use to directly or indirectly target platelets and their activation can offer better outcomes in patients who are at risk of developing cancer.

## Platelets and Immune Modulation

Among other functions, platelets contribute centrally to immune regulation. They interact with immune cells and participate in the innate and adaptive immune functions (132–136). Platelets initiate or modulate immune cell and wound sterilization responses along with vesicle-mediated transfer of surface proteins onto the immune cells in conjunction with stimulation (137, 138). Platelets also have high amounts of transforming growth factor-β (TGFβ), which is perceived as an immunosuppressive factor modulating T regulatory cell homeostasis (139, 140). Blocking TGF β1 receptor was shown to be beneficial in preventing ovarian cancer progression by the platelet-derived TGF β1 (141). Functional impairment of normal recognition and elimination pathways fosters development of a pro-tumorigenic microenvironment. Tumor immune surveillance and tumor cell-platelet cross talk may thwart immune cell recognition or recruitment of effector immune cells to tumors. The most intriguing reports show that platelets can directly suppress the immune cells that target or eliminate cancer cells. In one study, platelets inhibited and suppressed the function of CD8 as well as CD4 T cells mediated *via* TGFβ and lactate (55). In other studies, platelets were shown to protect tumor cells from natural killer (NK) cell cytotoxic activity by shielding them or by the transfer of MHC I onto the surface of tumor cells (142, 143). The importance of platelets becomes evident with respect to tumor immunity as platelets can not only modify immune cell responses and result in the silencing of the tumor targeted immunity but also modulate tumor cells by enveloping them or by conferring them a pseudonormal phenotype so they can go undetected under immune surveillance.

Among the many roles that platelets play in enriching the microenvironment for tumor cell survival, perhaps the most challenging to target may be the reversion of suppression on CD8^+^ T cells as platelets have been reported to inhibit CD8 T cell function (55). The CD8^+^ T cells have clinical relevance as their main function is to kill cancer cells and are predominant effectors in cancer immunotherapy (144). In CRC patients, the absence of activated CD8^+^ T cells within the tumor and tumor stroma predicted disease recurrence within 5 years, whereas for patients who do show presence of these T cells, predicts a long disease free survival (145). The predictive value of the T cell presence in the tumor has gained more importance in treatment response (146). If having platelets, especially those that have been educated by the tumor, within the tumor as well as in the microenvironment could potentially interfere with the T cell functioning, then targeting these tumor cell educated platelets could be a key consideration in managing cancer immunotherapy.

Another important factor in cancer immunotherapy is the expression of programmed death-ligand 1 (PD-L1) on the tumor cells. It is of significance as it interacts with and inhibits the cytotoxic CD8^+^ T cells by engaging with their surface programmed cell death 1 (PD-1) receptor (147, 148). PD-L1 therefore, is an indicative marker of immune suppression and is an important target in cancer immuno-oncology field. Recently, it was shown that PD-L1 expressed on non-tumor cells can also have an inhibitory effect on the cytotoxic CD8^+^ T cell responses against the tumor (149). Immune checkpoint inhibitors that inhibit the interaction of PD-L1 with immune cells has been widely used in clinical trials to relieve the immune cells of this tumor suppression (150–158). Therapy with this strategy alone using PD-L1 blocking antibodies showed a mixed response within the patient populations leading to the development of vaccines and T cell stimulatory molecules, or drug combinations along with PD-L1 inhibitors to improve the efficacy of immunotherapy by targeting more pathways that lead to tumor cell death and growth suppression (159–165). Several factors such as differential PD-L1 expression on tumors, negligible presence of tumor infiltrating lymphocytes, presence of other inhibiting marks on the immune cells, and other host factors could be contributing to the failure of response to anti-PD1/PD-L1 monotherapy. Moreover, the persistence of PD-L1 expression or the PD-L1 positive CTCs after anti-PD-L1 therapy in non-small cell lung cancer (NSCLC) patients correlated with progressive disease, suggestive of an escape mechanism to check point therapy (166). There could be some connection between unresponsive CTCs and platelet numbers in circulation as escape mechanisms for CTCs from immune surveillance could be achieved by being entrapped within platelet aggregates, or by expressing platelet proteins on their surface and masking themselves. Support for this notion comes from a study showing that patients who had higher platelet counts showed poorer response to PD-L1 therapy. The study showed that elevated platelet to lymphocyte ratio before treatment was associated with shorter overall survival and progression free survival in metastatic NSCLC patients who underwent nivolumab therapy (167). In light of these findings, the inhibition of platelets to boost or sustain immunotherapy responses is a highly viable option. Targeting platelets was shown to enhance adoptive T cell cancer therapy suggesting that platelet inhibition could lead to more durable immunotherapy responses. Genetically modified mice with dysfunctional platelets were used to model adoptive T cell therapy responses to demonstrate that platelets are restrictive to T cell mediated cancer immunotherapy and inhibiting platelet function could help improve immunotherapy response with active T cells (55).

## Connecting NSAIDs and Checkpoint Blockade

Interesting observations recently showed a positive correlation between COX-2 and PD-L1 responses in cancer cells (128). The upregulation of PD-L1 expression in tumor-infiltrating myeloid cells was also shown to be driven by COX-2 pathway (168). One of the major upstream targets of PD-L1 expression is hence presumed to be COX-2. COX-2 inhibition can therefore be explored to enhance the effect of immunotherapy. In other studies, COX-2 inhibition was used as an immunotherapy-enhancing tool. NSAID celecoxib, a COX-2 inhibitor, was shown to enhance the proliferation of NK T cells derived from laryngeal cancer patients (169). The inhibition of COX-2 was also shown to negatively impact immune evasion of tumor cells, as its inhibition was shown to synergize with PD-L1 blockade suggesting that COX inhibitors could be used in combination with immunotherapy (170). Other preclinical studies have also reported similarly that inhibiting cyclooxygenase pathway can have a synergistic effect with PD-L1 inhibitors (128). Aspirin and other NSAIDs that selectively target platelets, COX-2, or both could potentially be used as adjuvants with immunotherapy to impact the aberrant platelet driven or cyclooxygenase driven anti-immune tumor responses. Tumor heterogeneity and molecular subtypes, surface markers, microenvironment as well as chronic inflammation and other risk factors may sub-stratify patients for adjuvant treatment. In the case of CRC, these factors may segregate patients not only based on the type subtype but also by the kind of response they might show to therapy (171, 172). Microsatellite instability high status in CRC can also influence checkpoint blockade responses. For example, nivolumab provided durable responses and disease control in pretreated patients with dMMR/MSI-H metastatic CRC and could potentially be a new treatment option for these patients. Since inflammation is often associated with platelet activity these patients may also benefit from NSAID use (134). In order to understand which patient is most likely to benefit from a therapy or a combination of drugs, data from larger clinical trials and longer patient follow up is needed. Finally, aspirin or other NSAIDs recommendations to a patient will depend on their tumor consensus molecular profiling, platelet counts, along with their risks in developing adverse events. However, the available data from clinical trials suggests that the benefits of long-term aspirin use outweigh the risks in most cases.

## Conclusion

Platelets that primarily participate in normal wounding processes are also known to be involved in immune regulation, and tumor cell cross talk. Strong experimental and clinical evidence supports an active involvement of platelets in tumorigenesis and metastasis. Tumor-educated platelets have the potential to be used in cancer diagnostic and screening approaches. High platelet counts seem to correlate with poor treatment outcomes. The interference of platelets with immune cell functioning could be of importance in designing immunotherapy approaches with platelet inhibitors as adjuvants. The use of aspirin and other platelet-targeted drugs in inhibiting platelet function has been established in several clinical studies and can be readily administered to patients based on their disease state and platelet profile. Hence, targeting platelet–driven responses in enhancing the reactivation of cancer immunity appears to be a reasonable therapeutic strategy.

## Author Contributions

PKM, ML, AVS and DM wrote the manuscript. SK, MJO and DM edited and provided feedback on relevant sections to include in the manuscript.

## Conflict of Interest Statement

The authors declare that the research was conducted in the absence of any commercial or financial relationships that could be construed as a potential conflict of interest.
